# Stigmatization and Disparities in Healthcare Among LGBTQ Population in Africa: Advancing Health Equity

**DOI:** 10.1002/puh2.70185

**Published:** 2026-01-27

**Authors:** Ejovwokeoghene Joseph Omohwovo, Don Eliseo Lucero‐Prisno

**Affiliations:** ^1^ University of Port Harcourt Port Harcourt Nigeria; ^2^ Department of Global Health and Development Faculty of Public Health and Policy London School of Hygiene and Tropical Medicine London UK; ^3^ Office for Research, Extension and Innovations Bukidnon State University Malaybalay City Bukidnon Philippines; ^4^ Research and Innovation Office Biliran Province State University, Biliran Naval Philippines

## Abstract

With a population of over 1.5 billion people in 2024, Africa is home to a diverse group of individuals, including the Lesbian, Gay, Bisexual, Transgender, and Queer (LGBTQ) community. However, severe stigmatization and discrimination toward LGBTQ individuals have resulted in healthcare disparities. These disparities are exacerbated by limited access to medical care services, poor health outcomes, and a lack of cultural competence among healthcare providers. Thus, LGBTQ individuals are at a higher risk of sexually transmitted infections and mental health issues. This research commentary sheds light on the healthcare disparities faced by LGBTQ individuals in Africa and highlights the contributing factors, as well as the impact of stigmatization on their health. To tackle these disparities and promote healthcare equity for LGBTQ people in Africa, there is a need for increased advocacy for policy and legal reforms that protect their rights and encourage healthcare equity. Fostering education and awareness, among healthcare providers, policymakers, and the general public, and implementing efficient and sustainable data‐collection strategies to address their healthcare needs, is also crucial. Hence, a concerted effort is essential to foster collaboration among various stakeholders, including healthcare providers, non‐governmental organizations (NGOs), government health agencies, and policymakers, to advance inclusive healthcare equity for the LGBTQ population in Africa.

## 1 Introduction

Health equity, which ensures equal access to quality medical care, is a fundamental right for all individuals worldwide, regardless of their race, ethnicity, gender, sexual orientation, or socioeconomic status [1]. However, lesbian, gay, bisexual, transgender, and queer (LGBTQ) individuals, especially youth, often face significant healthcare inequalities stemming from minority stress, stigmatization, victimization, heterosexism or heteronormativity, and discrimination [2]. This issue is particularly severe in Africa, with a population of over 1.5 billion people in 2024 [3], as the continent is home to a diverse people with different cultural and social backgrounds, including the LGBT community, but lacks adequate policies that protect the rights of LGBTQ individuals as well as meeting their healthcare needs, thus limiting their access to adequate healthcare services.

LGBTQ individuals, including those in Africa, are at a higher risk of sexually transmitted infections (STIs) such as syphilis, HIV/AIDS, gonorrhea, Herpes simplex virus, and Human papillomavirus [4, 5], as well as mental health issues [6]. The existence of disparities in the healthcare system in Africa toward LGBTQ individuals exacerbates public health challenges in the region [7, 8]. For instance, according to a study conducted in Ghana, the LGBT population in the country faces several disparities in health, including behavioral, physical, and mental health issues [9].

Addressing disparities in the medical care system is crucial to ensure an equitable healthcare system for all individuals, especially the LGBTQ population in Africa [7]. This will enhance the overall healthcare outcomes and pave the way for a more inclusive and accessible healthcare system in the region. This research commentary sheds light on the healthcare disparities faced by LGBTQ individuals in Africa, highlights the impact of stigmatization and discrimination on their health, and suggests successful interventions to promote healthcare equity. Implementing these initiatives will strengthen and cultivate an equitable and inclusive healthcare system that prioritizes the health needs of the LGBTQ population in the region.

### 1.1 Contributing Factors to Stigmatization and Discrimination Within Healthcare Facilities Against LGBTQ Individuals in Africa

Across many African countries, LGBTQ individuals face significant challenges in accessing healthcare, mainly due to the increasing stigma and discrimination they encounter from individuals and institutions, including in medical settings. This is often based on the fact that people and institutions in Africa hold beliefs and practices that foster a hostile environment for the LGBTQ population [10]. Several indigenous factors exacerbate the issues of inequality and disparities in healthcare settings for LGBTQ individuals in Africa, emphasizing the urgent need to highlight the significant contributing circumstances in the region.

Societal and cultural norms, rooted in heteronormative values and prejudices against nonconforming sexual orientations and gender identities, contribute significantly to the persistent marginalization of LGBTQ individuals within medical care settings in many African countries [11]. These norms often foster an environment of stigma and discrimination, deterring LGBTQ individuals from accessing healthcare services without fear of judgment or rejection. Moreover, healthcare providers may internalize and perpetuate societal and even cultural prejudices as they pertain to gender identity and sexual orientation, leading to discriminatory behavior toward LGBTQ individuals [11].

Another notable contributing factor is the persistent promotion of anti‐LGBTQ+ legislation, often driven by polarizing political discourse from leaders throughout Africa. In their efforts to gain support and divert attention from their own shortcomings, certain African politicians characterize LGBTQ+ identities as an external influence that jeopardizes social harmony and undermines societal values and ethics [4]. Specifically, in many African countries during election periods, politicians often advocate for stricter anti‐LGBTQ+ measures [4]. This rhetoric fosters hostility among their supporters and others toward LGBTQ+ individuals, even impacting the health sector and frequently resulting in stigmatization and discrimination against these communities.

Institutional barriers in healthcare facilities in several African nations also pose a significant factor that perpetuates stigma and discrimination against LGBTQ individuals in medical care settings [12]. Without policies and practices tailored to the specific health needs of the LGBTQ community, LGBTQ individuals may encounter a hostile environment, leading to stigmatization and discrimination [12].

Religious beliefs that condemn same‐sex relationships also pose as a contributing factor to discrimination against LGBTQ individuals in healthcare facilities across many African countries. Conservative religious beliefs, often based on Christianity and Islam, promote anti‐LGBTQ attitudes and may cause some healthcare workers to put their beliefs above patient care. This can lead to refusal of services or discriminatory treatment, amplifying discrimination against LGBTQ individuals in healthcare settings. For instance, a study found that religious affiliation is closely linked to negative attitudes toward LGBTQ individuals, with religiosity particularly heightening these attitudes among Christian and Muslim practitioners [13].

### 1.2 Impact of Stigmatization and Discrimination on LGBTQ Health in Africa

The impact of marginalization and prejudice against the LGBTQ population in healthcare varies significantly across regions. Although Western countries tend to prioritize healthcare and protections for LGBTQ individuals, in regions like Africa, Eastern Europe, and parts of Asia, LGBTQ individuals still face substantial stigma and discrimination tied to long‐held cultural, religious, and legal issues. The narratives about stigma and discrimination against the LGBTQ community in many African countries resonate with non‐Western states that have collectivist cultural traits [10]. However, in many African countries, these issues are especially severe, marked by rising restrictive laws and widespread societal stigma that greatly marginalize LGBTQ people, even within healthcare settings. Such practices create significant barriers to healthcare access, adversely affecting the health and well‐being of LGBTQ individuals across the continent. Therefore, it is imperative to highlight the serious consequences of stigmatization and discrimination in healthcare settings, which disproportionately impact the LGBTQ population in numerous African countries.

Societal stigmatization and discrimination, often influenced by religious and cultural beliefs, against LGBTQ individuals are pervasive in many nations in Africa [11] and have significant impacts on their physical and mental health [6, 7]. Oftentimes, negative attitudes, prejudice, and stereotypes result in the exclusion and marginalization of LGBTQ individuals from society [12]. Moreover, this stigma and discrimination often lead to social isolation, rejection, and even violence against LGBT individuals. For instance, in Ghana, a study revealed that LGBTQ participants who shared their experiences within their families, the LGBTQ+ community, and the broader public faced significant stigmatization, with many families disowning their LGBTQ members. In addition, the participants highlighted that the pervasive discrimination that they faced contributed to poor mental health outcomes for many, as individuals struggled with the emotional toll of rejection [14].

The stigmatization and discrimination of LGBTQ individuals extend beyond society to healthcare settings [15]. The LGBTQ population frequently experiences discriminatory practices, including refusal of care, judgmental attitudes, and lack of cultural competence, from healthcare providers [15]. Thus, it creates a hostile environment where LGBTQ individuals may feel ashamed and unsafe or hesitant to seek necessary medical care.

The stigmatization and discrimination faced by the LGBTQ population in healthcare facilities in Africa have severe health consequences that may negatively impact their overall well‐being [12], as it may discourage them from accessing essential healthcare services, such as preventive care, early diagnosis, and appropriate treatment for physical health conditions [12]. Moreover, their mental health can also be significantly impacted, leading to increased rates of depression, anxiety, substance abuse, and even suicidal thoughts [7].

### 1.3 Barriers to Accessing Healthcare for LGBTQ Individuals in Africa

In many African countries, the prevalent lack of education and awareness regarding the unique healthcare needs of LGBTQ individuals has contributed to continual discrimination and stigma against this community [16]. According to Ref. [13], the global healthcare landscape, including Africa, is grappling with a significant gap in LGBTQ+ care, where many medical providers and staff lack sufficient knowledge and training to address the unique health needs of LGBTQ+ individuals. Healthcare providers may lack the necessary skills and knowledge to provide adequate care for LGBTQ individuals, resulting in disparities in healthcare access. A study in South Africa highlighted significant gaps in the literature, practice guidelines, and policies regarding LGBTQ healthcare, including the exclusion of LGBTQ issues from healthcare worker curricula [16]. Thus, LGBTQ individuals may feel uncomfortable or unsafe disclosing their sexual orientation or gender identity to healthcare providers, creating an environment that hinders optimal care. For instance, according to Ref. [17], healthcare professionals in South Africa do not have sufficient training to manage the LGBT population seeking medical care services. The lack of adequate knowledge among healthcare providers regarding the unique needs and sensitivity required to provide appropriate care for LGBTQ individuals can inadvertently perpetuate stigma and discrimination within the healthcare system, resulting in withdrawals from healthcare services. In Kenya, due to stigmatization in healthcare services, there were significant dropouts from HIV prevention programs among transgender women and gay men with HIV infections [18].

The criminalization of same‐sex relationships in many African countries is also a significant barrier to healthcare access for LGBTQ individuals, exacerbating the healthcare disparities they face [19]. The existence of punitive laws prohibiting same‐sex relationships in several African countries fosters a pervasive fear of persecution among LGBTQ individuals, preventing them from accessing healthcare services and deterring providers from offering care, thus cultivating a culture of fear that leads many LGBTQ individuals to hesitate or refuse to disclose their sexual orientation to physicians and consequently delay seeking healthcare, potentially resulting in poor health outcomes and an increased reliance on unsafe practices. Moreover, the absence of policies in many African countries that consider the health needs of LGBTQ individuals and protect their rights results in their marginalization and discrimination within healthcare facilities, making it even more challenging for them to access appropriate healthcare services.

The financial struggles faced by many individuals in several African nations, including the high costs of accessing healthcare, pose significant barriers for the LGBTQ population in Africa [12]. Societal discrimination often leads to economic challenges that prevent many LGBTQ individuals from accessing necessary healthcare services, including gender‐affirming care and routine checkups [12]. Additionally, many LGBTQ individuals find it difficult to afford basic necessities, let alone healthcare services [12]. Consequently, the significant economic instability in several African countries forces the LGBTQ community to prioritize immediate needs over medical care. The lack of financial resources and support leaves LGBTQ individuals vulnerable to health complications, exacerbating existing health disparities within this population.

### 1.4 Efforts Toward Equity in Africa

Several organizations, including non‐governmental organizations (NGOs), have implemented community‐based interventions in various African countries to enhance training and awareness among healthcare providers. For instance, in South Africa, the Triangle Project, in collaboration with other organizations, has organized multiple training sessions and campaigns for healthcare workers to address the mental and physical needs of the LGBTQ population [20].

To promote the rights of LGBTQ individuals as well as protect them from discrimination, policy and legal reforms have been implemented in some African countries. In 2019, Angola decriminalized same‐sex relationships and banned discrimination on the basis of sexual orientation [21]. Similarly, in 2020, Gabon followed suit by criminalizing discrimination on the basis of sexual orientation and gender identity [22].

In addition to national efforts, international organizations, such as the World Health Organization (WHO) and the Centers for Disease Control and Prevention (CDC), have played a crucial role in expanding healthcare access for LGBTQ individuals in Africa. The United States Agency for International Development (USAID) has also funded several programs to improve equitable access to healthcare for LGBTQ individuals in several African countries, including Côte d'Ivoire, Kenya, Malawi, Namibia, South Africa, and Nigeria [23]. These efforts have been instrumental in addressing the healthcare disparities faced by the LGBTQ population in many countries in Africa and promoting healthcare equity.

Despite these national and international efforts to protect the rights of the LGBTQ population and ensure equitable healthcare access, significant disparities persist in healthcare access for LGBTQ individuals in Africa. As of 2024, of the 54 countries in Africa, 31 have criminalized same‐sex relationships, and only a few, including Angola, South Africa, Mauritius, and Botswana, have laws protecting against discrimination on the basis of sexual orientation [24]. Additionally, stigma and discrimination against LGBTQ individuals remain prevalent in many African countries.

### 1.5 Future Directions

To address these disparities and promote healthcare equity for LGBTQ individuals in Africa, the following are recommended:

First, fostering education and awareness among healthcare providers, policymakers, and the general public is vital to reducing the high burden of stigma and discrimination against LGBTQ individuals in Africa. Hence, policies that promote community‐based interventions that meet the specific needs of LGBTQ individuals, including enhanced training programs for healthcare providers and public awareness campaigns, should be implemented in the region. This will increase awareness and reduce stigma and discrimination against LGBTQ individuals.

Second, to ensure the protection of LGBTQ individuals' rights in Africa, it is essential to include them in the advocacy for policy and legal reforms that safeguard their rights and promote healthcare equity. Thus, including LGBTQ individuals in anti‐discrimination laws and developing policies that advance healthcare equity is crucial; this will strengthen advocacy for the decriminalization of same‐sex relationships in several African countries.

Third, to promote health equity for LGBTQ individuals in Africa, implementing effective data‐collection strategies on their healthcare needs, challenges, and experiences is essential to developing targeted interventions. Hence, investments in research, focused on the healthcare needs and experiences of LGBTQ individuals in Africa, should be made to inform evidence‐based interventions. Additionally, a concerted effort involving collaboration among various stakeholders, such as government health agencies, NGOs, religious bodies, international organizations, and healthcare providers, should be encouraged in Africa. This will ensure that sufficient funds and resources are allocated to create inclusive healthcare spaces, provide targeted health services, and address the economic challenges faced by LGBTQ+ individuals. Moreover, building strong support systems within the LGBTQ community in several African nations is crucial for fostering sustainable resilience. This can be achieved through initiatives that offer mental health resources, counseling services, safe community spaces, and allyship programs, providing a safety net for LGBTQ individuals and promoting overall well‐being [25]. A study in Ghana highlighted that LGBTQ+ participants who experienced rejection from their families and communities asserted to have garnered significant support within the LGBTQ+ community, helping them to cope with the stigma and emotional challenges associated with their sexual orientations [14].

Lastly, lawmakers in several African countries should enact laws that prohibit the use of inflammatory language against LGBTQ+ individuals. This measure would prevent individuals, including politicians, from exploiting anti‐LGBTQ+ sentiments to gain followers. Moreover, it is crucial to include LGBTQ+ individuals, including advocates, in media and public discussions to ensure their representation, as well as to counteract harmful comments about the LGBTQ+ community and hold individuals, particularly politicians, accountable for using anti‐LGBTQ+ rhetoric to bolster their own influence.

## 2 Conclusion

The healthcare disparities faced by the LGBTQ population in Africa represent a significant public health challenge that requires urgent attention. Stigmatization, discrimination, and various barriers to healthcare access have severe consequences for their physical and psychological well‐being. Although several organizations have made notable strides in raising awareness, promoting inclusivity, and advocating for the rights of LGBTQ individuals in Africa, significant barriers persist. Overcoming these challenges requires a concerted and continuous effort by various stakeholders. Moreover, African countries should create a more inclusive and equitable healthcare system by implementing policy reforms and addressing the unique healthcare needs of the LGBTQ population.

## Conflicts of Interest

The authors declare no conflicts of interest.

## Data Availability

Data sharing not applicable to this article as no datasets were generated or analyzed during the current study.
